# Risk-Based Monitoring in Clinical Trials: 2021 Update

**DOI:** 10.1007/s43441-022-00496-9

**Published:** 2023-01-09

**Authors:** Amy Adams, Anina Adelfio, Brian Barnes, Ruth Berlien, Danilo Branco, Amanda Coogan, Lauren Garson, Nycole Ramirez, Nicole Stansbury, Jennifer Stewart, Gillian Worman, Paula Jo Butler, Debby Brown

**Affiliations:** Association of Clinical Research Organizations (ACRO), 601 New Jersey Ave NW #350, Washington DC, 20001 USA

**Keywords:** Risk-Based Monitoring, Risk-Based Quality Management, Centralized Monitoring, Clinical Trial Quality, RBM, RBQM

## Abstract

Clinical trial quality depends on ensuring participant safety and data integrity, which require careful management throughout the trial lifecycle, from protocol development to final data analysis and submission. Recent developments—including new regulatory requirements, emerging technologies, and trial decentralization—have increased adoption of risk-based monitoring (RBM) and its parent framework, risk-based quality management (RBQM) in clinical trials. The Association of Clinical Research Organizations (ACRO), recognizing the growing importance of these approaches, initiated an ongoing RBM/RBQM landscape survey project in 2019 to track adoption of the eight functional components of RBQM. Here we present results from the third annual survey, which included data from 4889 clinical trials ongoing in 2021. At least one RBQM component was implemented in 88% of trials in the 2021 survey, compared with 77% in 2020 and 53% in 2019. The most frequently implemented components in 2021 were initial and ongoing risk assessments (80 and 78% of trials, respectively). Only 7% of RBQM trials were Phase IV, while the proportions of Phase I–III trials ranged 27–36%. Small trials (< 300 participants) accounted for 60% of those implementing RBQM. The therapeutic areas with the largest number of RBQM trials were oncology (38%), neurology (10%), and infectious diseases (9%). The 2021 survey confirmed a pattern of increasing RBM/RBQM adoption seen in earlier surveys, with risk assessments, which have broad regulatory support, driving RBQM growth; however, one area requiring further development is implementation of centralized monitoring combined with reductions in source data verification (SDV) and source data review (SDR).

## Introduction

Clinical trials are constantly evolving in response to advances in technology, new methods of data analysis, and emerging trends in trial design, such as decentralized clinical trials (DCTs). One of the major developments in clinical trial management in recent years is implementation of the risk-based monitoring (RBM) and risk-based quality management (RBQM) frameworks [1–4]. Adoption of these approaches is increasing due to their efficiency, regulatory support, and enhancement of trial quality [5–7]. In addition, RBM/RBQM uptake has dramatically accelerated since 2020, most likely due to the expansion of off-site trial activities such as centralized and remote monitoring during the COVID-19 pandemic.

In 2019, the Association of Clinical Research Organizations (ACRO), having observed the evolution in clinical trial execution to harness new technological advances, set out to quantify the adoption of RBM/RBQM. ACRO’s membership, which includes global contract research organizations (CROs) and technology companies, is uniquely positioned to provide data on clinical trial management to support advocacy, policy development, and stakeholder education.

To measure the uptake of new approaches to trial monitoring and management, ACRO initiated an ongoing landscape survey of RBM/RBQM implementation across thousands of clinical trials conducted by ACRO members [8]. This long-term initiative has produced articles describing the first two annual surveys, covering the years 2019 and 2020, with results from the third annual survey covering 2021 reported here [8, 9]. ACRO plans to release future annual reports and other resources produced by the landscape survey project as part of the organization’s mission to support the continued adoption of emerging advances in clinical trial management.

**Figure 1:**
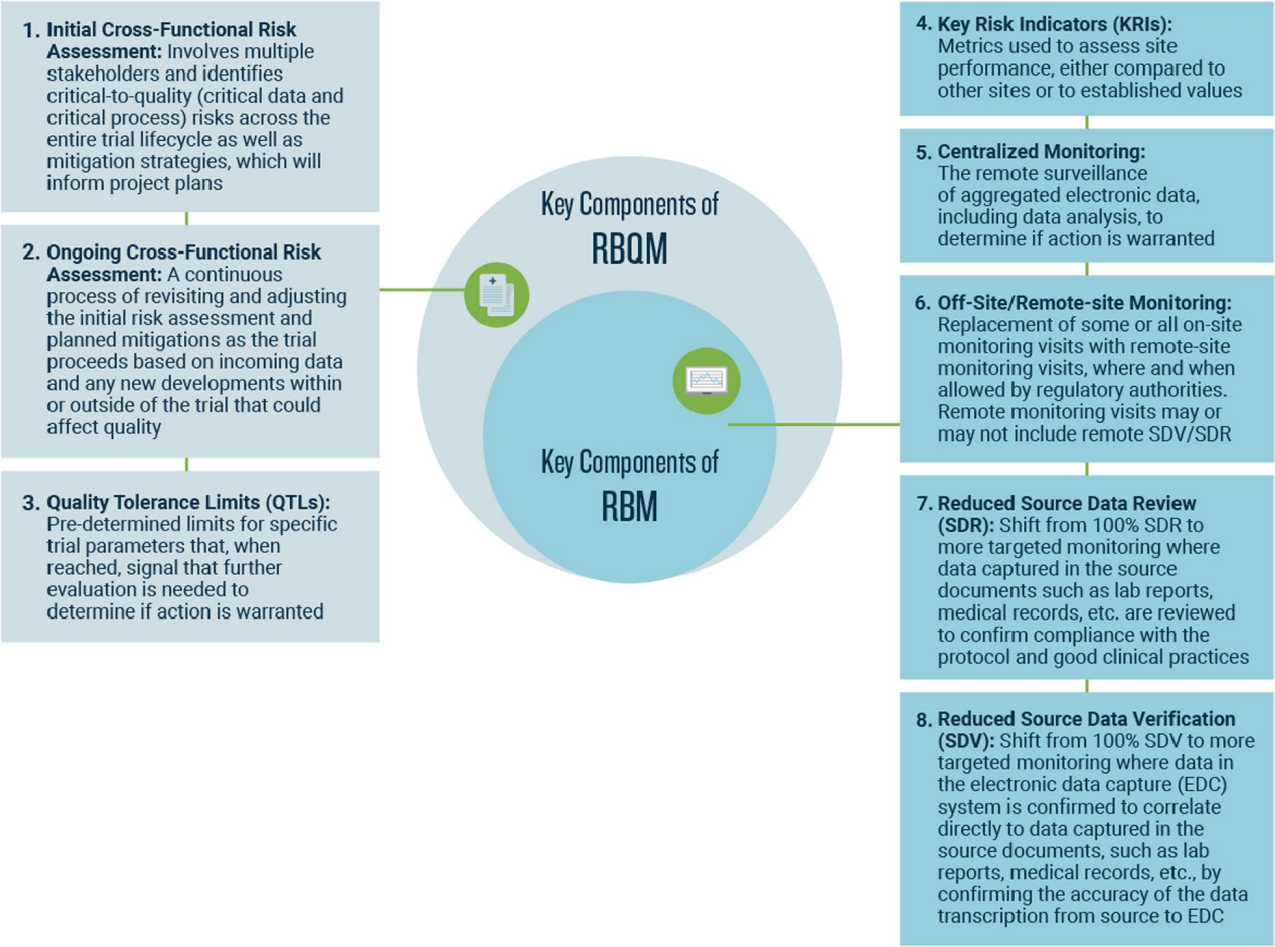
Legend: Component definitions were developed by ACRO member representatives during initial planning for the RBM/RBQM landscape survey. Components 1–3 are the “backbone” of the RBQM framework, acting as the foundation for a wide range of trial activities beyond just monitoring. The RBM framework consists of Components 4–8, which include tools and activities specific to monitoring. Figure content adapted from Barnes et al. [8].

The first landscape survey publication covered results from 2019 to the first half of 2020, using the 2020 data to capture changes in trial monitoring at the onset of the pandemic [8]. That article presented the clearest definition, to date, of RBM and its place within the larger RBQM framework, which consists of eight functional components: an initial cross-functional risk assessment, an ongoing cross-functional risk assessment, quality tolerance limits (QTLs), key risk indicators (KRIs), centralized monitoring, off-site/remote-site monitoring, reduced source data verification (SDV), and reduced source data review (SDR). The first three components are specific to RBQM, while the remaining five comprise RBM. Figure 1 presents definitions—formulated prior to launching the first landscape survey—for all eight components.

A subsequent article reporting full-year landscape survey data for trials ongoing during 2020 has since been published, describing a continuing trend of increased RBM/RBQM adoption [9]. The data highlighted the importance of four components that are particularly critical to increasing future uptake of RBM/RBQM: initial risk assessment, ongoing risk assessment, centralized monitoring (ideally combined with reduced SDV/SDR), and QTLs.

ACRO’s third annual RBM/RBQM landscape survey covering trials ongoing in 2021 extends our examination of adoption rates and implementation trends. This article explores survey data from 2019 to 2021 and identifies the components that must increase in implementation in to order to realize the full potential of these frameworks.

## Methods

The RBM/RBQM landscape survey methodology has been described previously [8, 9]. In this survey update, seven ACRO members provided data on clinical trials ongoing as of December 31, 2021, for which those companies provided project management and/or clinical monitoring services. A neutral, third-party vendor collected and analyzed the aggregated data in a blinded fashion. Companies participating in the survey have varied from year to year—reflecting company changes within ACRO’s membership.

Information on RBQM component implementation was provided for 4889 trials ongoing in 2021, 1270 of which were new studies started that year. Table 1 provides a comparison of the data sets from 2019, 2020, and 2021.

**Table 1:** Summary of Clinical Trials included in the ACRO RBM/RBQM Landscape Survey 2019–2021

	2019	2020	2021
CROs participating	7	6	7
Number of trials	6513	5987	4889
New study starts	709	908	1270

## Results

### Data Set Compared to Previous Years

The most recent 2021 RBM/RBQM landscape survey included 4889 clinical trials, a reduction compared to 2019 and 2020 (Table 1). The 1270 new study starts accounted for 26% of 2021 ongoing trials, whereas only 15% of 2020 trials were new study starts.

The number of trials without any RBQM components totaled 3469, 1272, and 594 in 2019, 2020, and 2021, respectively. The year-over-year reductions in these trials is expected since more operational teams are adopting risk-based practices. It is possible, however, that a small number of traditional trials were initiated in 2021.

#### Implementation of RBM/RBQM

**Figure 2:**
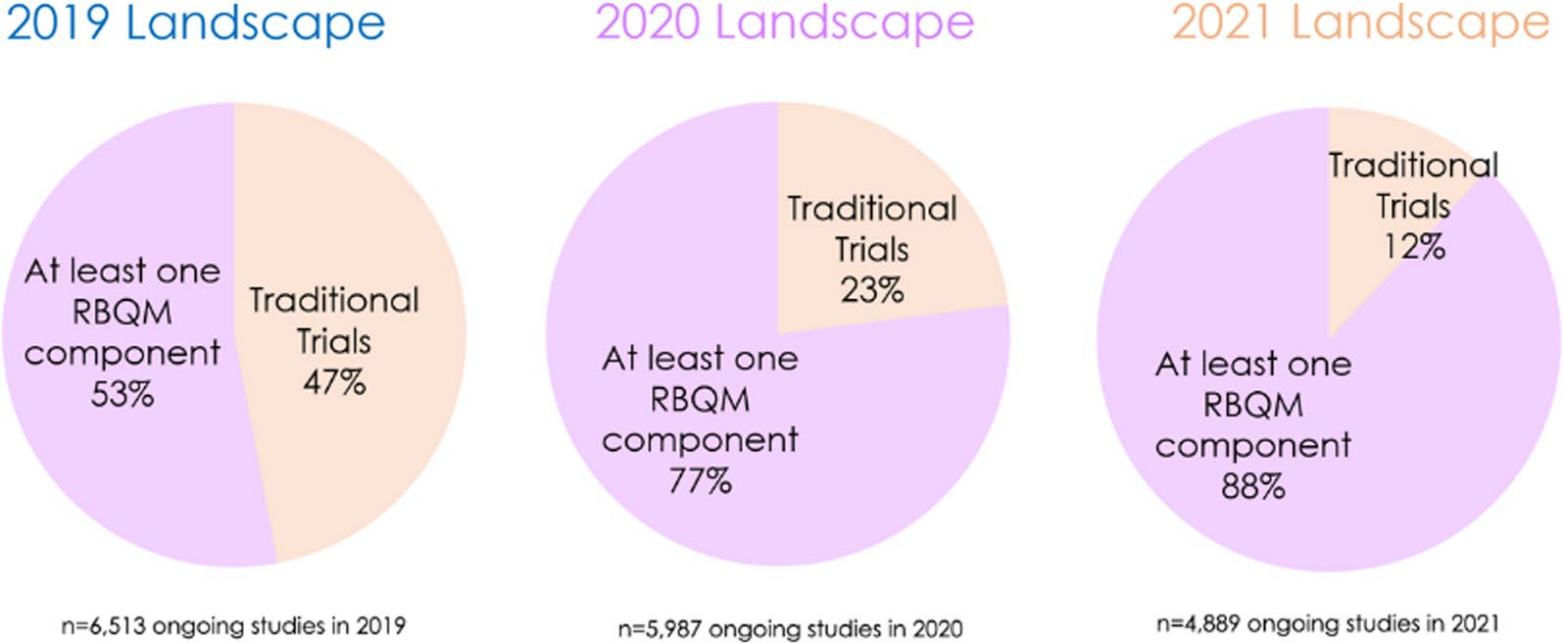
Legend: The percentage of ongoing clinical trials with at least one RBQM component grew each year, resulting in a 35 percentage-point increase from 2019 to 2021. The completion of multi-year traditional trials and greater adoption of RBQM in new study starts both contributed to the increase.

The vast majority of 2021 trials (88%) included at least 1 RBQM component, an increase from 53% of trials in 2019 and 77% in 2020 (Fig. 2). Despite the differences in the yearly data sets, the large number of ongoing trials each year supports a trend of increasing adoption of RBM/RBQM. These data also illustrate the completion of traditional trials with no RBQM components—which accounted for nearly half of all ongoing trials in 2019, but merely 12% in 2021—as well as broad adoption of one or more RBQM components in new study starts.

**Figure 3:**
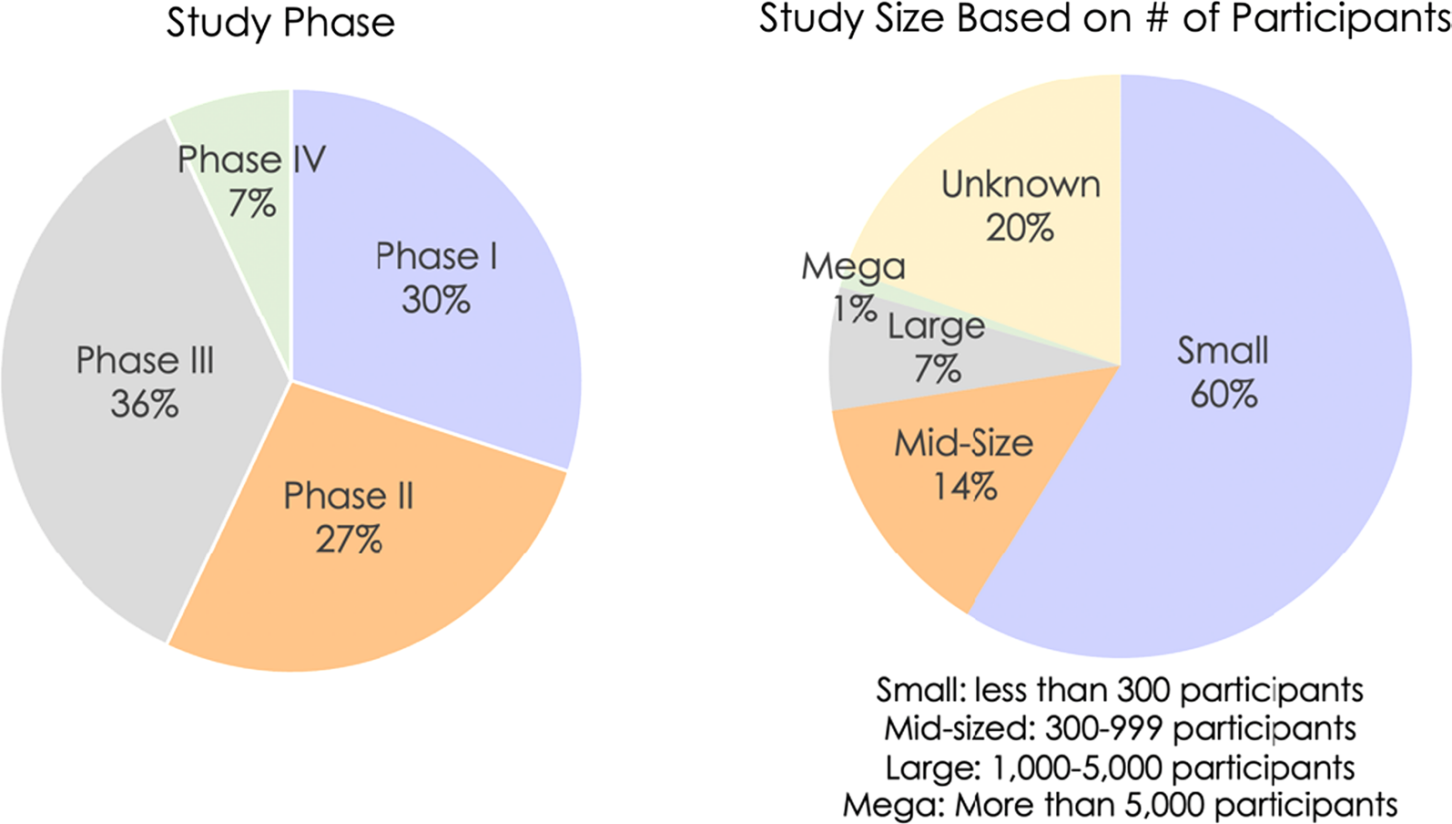
Legend: Of the 88% or 4303 trials included in the 2021 landscape survey that implemented at least one RBQM component, 93% were Phase I–III. The percentages of small, mid-sized, large, and mega-sized trials is roughly consistent with the breakdown by phase, as Phase III and IV trials tend to be larger, and Phase I and II trials tend to be smaller.

Of the 88% of 2021 trials implementing at least one RBQM component, the percentages of Phase I, Phase II, and Phase III trials were similar, ranging 27–36% (Fig. 3). The lower percentage (7%) of Phase IV studies, which often enroll thousands of participants, may reflect a smaller overall percentage of these trials in the data set, the longer duration of these trials, and/or different regulatory expectations for monitoring of approved therapies. Correspondingly, only 8% of RBQM trials were large- or mega-sized trials enrolling > 1000 participants. The percentage of small RBQM trials enrolling < 300 participants (60%) was similar to the combined percentage of Phase I and II trials (57%), which tend to be smaller than Phase III and Phase IV trials. The fact that study size was unknown for 20% of the trials in the 2021 data set suggests that these results should be interpreted with caution; however, the data do indicate that RBQM is currently being implemented in a variety of study types.

### Therapeutic Area

RBQM is implemented in clinical trials across a wide range of therapeutic areas, with oncology (38%), neurology (10%), and infectious diseases (9%) trials accounting for the largest percentages of RBQM trials in the 2021 landscape survey. Also of note, 4% of RBQM trials in the 2021 data set investigated COVID-19 vaccines or treatments and therefore must have been initiated in 2021 or 2020. It is possible that oncology trials are overrepresented in the present analysis because these trials tend to be highly complex and may be more likely to be outsourced to CROs. The proportions of the top 3 therapeutic areas in this data set are, however, roughly consistent with those reported in an analysis of > 185,000 clinical trials conducted from 2000 to 2015 [10].

### Implementation of Individual RBQM Components

**Figure 4:**
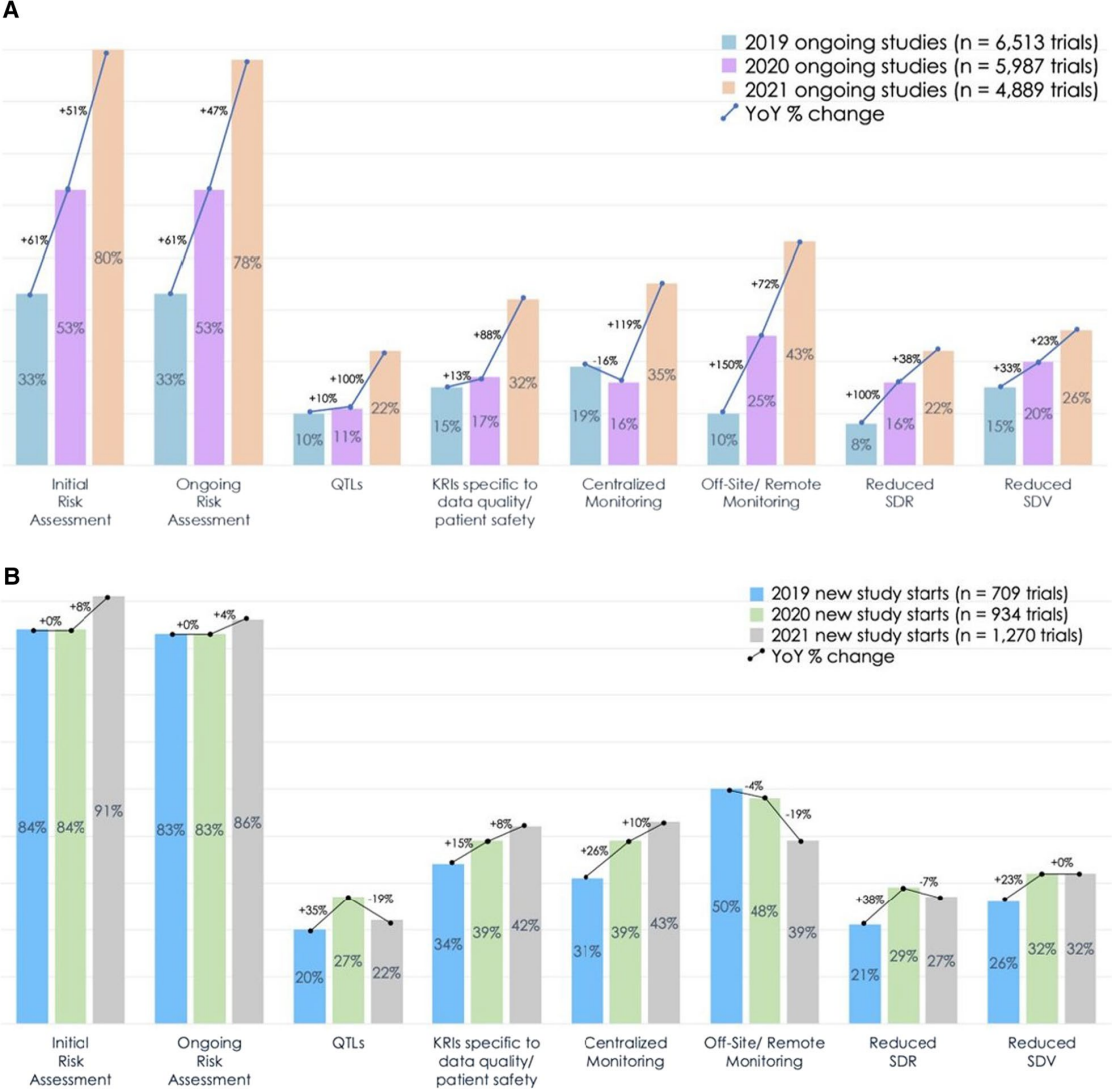
Legend: (**A**) Prevalence of individual RBQM components, by year, for all trials in the 2019, 2020, and 2021 data sets. Implementation of all components increased from 2019 to 2021. (**B**) Prevalence of individual RBQM components, by year, for new study starts each year from 2019 to 2021. A less consistent pattern for year-over-year changes in component implementation was seen for new study starts versus all trials.

Most trials implementing RBQM do not utilize every component of that framework. The choice of which components to use in a given clinical trial may be based on trial design and other factors, such as sponsor preference or engagement of a CRO. As seen in previous years of the landscape survey, component adoption rates vary; therefore, we assessed the prevalence of each of the eight RBQM components for each year of the landscape survey (Fig. 4).

For both new study starts and trials initiated in years prior, implementation increased for all components from 2020 to 2021 (Fig. 4A). This was consistent with the increases seen from 2019 to 2020 for every component except centralized monitoring, which fell slightly from 19 to 16% of studies, although implementation of this component then rebounded, increasing to 35% of studies in 2021. Initial and ongoing risk assessments were implemented in a higher percentage of trials compared with the other six components for each of the three survey years.

Different trends in component implementation were seen when looking at only new trials implemented each year of the survey (Fig. 4B). New study starts generally had higher rates of component implementation but smaller year-over-year percentage increases compared with total trials (Fig. 4A). Even higher percentages of new studies starting in 2021 included risk assessments, indicating alignment of risk assessment activities to regulatory guidance. Centralized monitoring increased steadily year-over-year for new study starts from 31% in 2019 to 43% in 2021, in contrast to the pattern seen for total studies (Fig. 4, A and B). Particularly notable is the discrepancy in centralized monitoring implementation between new study starts (39%) versus total studies (16%) during 2020, the first year of the pandemic. The smallest increases from 2020 to 2021 were seen for reduced SDV and reduced SDR, supporting previous observations that adoption of these components is lagging and that 100% SDV/SDR is still being relied on too frequently.

### Effect of Trial Size and Phase on RBQM Implementation

RBQM component implementation in 2021 varied by trial size (data not shown). Implementation of individual components tended to be less for small- to mid-sized trials and greater for large- and mega-sized trials. This may reflect greater utility and cost–benefit for RBQM implementation in larger trials with well-defined critical data and processes as opposed to smaller trials, which are more likely to be early-phase studies.

Initial and ongoing risk assessments were implemented in a higher proportion of trials (80–90% and 72–90%, respectively), regardless of size, compared with other components. More frequent implementation of KRIs, centralized monitoring, and off-site remote monitoring in Phase III trials compared to the other phases are positive trends related to trial type and enrollment. These components would be expected to have a greater impact in larger experimental trials of unapproved therapies.

## Discussion

The 2021 ACRO landscape survey provides a clear picture of RBM/RBQM adoption in clinical research. These data confirm the results of previous surveys, while also highlighting missed opportunities and areas for improvement. In addition, the increasing number of new studies starting year-over-year provides a better lens for examining the most recent trends in clinical trial practices.

### Rethinking the Metrics

Due to the generally low adoption of RBM/RBQM in 2019, we used the percentage of trials with at least one RBQM component as the primary metric of implementation. The thinking was that increases in this metric over time would indicate successful RBQM adoption. The increase in trials with at least one RBQM component from 53% in 2019 to 88% in 2021 shows both major progress in RBQM adoption and the limitations of this measure for future analyses now that most clinical trials have at least one component. In addition, our 2021 data show that 80% of trials implemented at least the initial risk assessment and 78% implemented the ongoing risk assessment, while the implementation of the other six components ranged from 22 to 43%. This makes it more difficult to assess changes in the frequency of other components based on the “at least one RBQM component” metric alone.

It is now clear that the implementation of individual components and, in the future, combinations of components should be analyzed to identify new RBM/RBQM adoption trends. This conclusion is also consistent with our qualitative observations of evolving attitudes toward these frameworks—specifically that more trials are now implementing two or more components.

### Risk Assessment Leads the Way

Risk assessments identify critical-to-quality (CtQ) factors—those data and processes that support the primary safety and efficacy endpoints and the overall trial objectives. Initial risk assessments are conducted during protocol development, and they determine the initial monitoring strategies. Ongoing risk assessments are reassessments of performance with the intention to make any necessary adjustments to the monitoring plan or strategy. Sponsors and investigators can allocate mitigation resources more effectively by targeting CtQ factors for monitoring instead of other factors that are not likely to compromise the trial. For this reason, it makes sense that the initial and ongoing risk assessments are the RBQM components with the highest adoption rate.

Despite the progress made in risk assessment implementation, one area for improvement is the timing of the *initial* risk assessment. Typically, these assessments are done after the protocol is finalized—but the most value is gained by beginning the initial assessment before protocol development, though it may be revised or repeated during and after the protocol is completed. This timing is consistent with recent Medicines and Healthcare products Regulatory Agency (MHRA) guidance, which recommends that a risk assessment “be done as early as possible” and indicates that inspectors review risk assessments whenever risk-adapted approaches are used in a clinical trial [7]. The MHRA guidance has had a strong impact on trial sponsors’ acceptance of risk assessments.

Risk assessments support not only the assessment and development of monitoring strategies during protocol development but also operational monitoring post-protocol development. This is important for all trials, but is especially important for complex trials such as DCTs, which incorporate telemedicine or digital health technologies and have trial activities that take place outside traditional trial sites [11]. For example, the use of local laboratories requires collecting reference ranges from each, which takes a significant amount of time and effort. A risk assessment can help determine which laboratory tests are necessary to the trial’s safety and efficacy endpoints and whether they are best done locally or at a central laboratory. Another challenge in modern clinical trial management is the increasing emphasis on inclusion and diversity during trial enrollment. Failure to enroll a representative participant population presents unique risks to the validity and acceptance of trial results. Potential problems can be identified by a risk assessment and then addressed by mitigation strategies defined during protocol development.

The International Council for Harmonisation of Technical Requirements for Pharmaceuticals for Human Use (ICH) Guideline for Good Clinical Practice (GCP) E6(R2) recommends that all trials incorporate, at minimum, the three RBQM-specific components: an initial cross-functional risk assessment, an ongoing cross-functional risk assessment, and quality tolerance limits (QTLs) informed by the risk assessments [12]. ICH E6(R2) and other guidance from regulatory bodies reflect the fact that risk assessments are foundational not only to QTLs but also to the development and execution of the five components that define RBM: KRIs, centralized monitoring, off-site/remote-site monitoring, reduced SDV, and reduced SDR [3, 4, 7, 8].

### Increasing Centralized Monitoring—the Next Step

Functionally, centralized monitoring is the component with the greatest potential to enhance the benefits of RBM. In addition to enabling flexible execution of site monitoring (on-site or remote, depending on findings), centralized monitoring can identify—in near real-time—protocol deviations, gaps in adverse event reporting, and safety concerns. Centralized monitoring allows for aggregation of data from multiple sources (e.g., electronic data collection, electronic participant-reported outcomes, eDiaries, laboratory tests, and wearable devices) and different trial sites. These disparate data streams can then be visualized together, providing a more complete picture for better detection of inconsistencies and anomalies.

Another advantage of centralized monitoring is its ability to detect differences in the range, consistency, and variability of data within or across sites. These trends are reviewed for systematic or significant errors in collection and reporting, as well as potential data manipulation or data integrity problems. Site characteristics and performance metrics should likewise be tracked by centralized monitoring, with anomalies, such as those defined by KRIs and QTLs, triggering site monitoring for specific sites or processes. This should be in conjunction with further ongoing risk assessments and any adjustments to a monitoring plan as needed.

Centralized monitoring also allows clinical research associates (CRAs) to focus on the on-site sampling of critical data and monitoring activities that can only be performed on-site, such as confirmation of participant eligibility or assessment of protocol adherence, GCP, and other regulatory requirements. A combination of site monitoring and centralized monitoring allows for faster, reliable, and more efficient detection of potential adverse events and other issues affecting trial quality.

The emergence of the COVID-19 pandemic in 2020 was expected to increase the use of centralized monitoring, as it did remote monitoring, but this was not the case [8, 9]. However, the percentage of new studies starting that year that included centralized monitoring was more than twice that for all 2020 trials, which indicates a very recent increase in uptake, likely due to the pandemic. The use of centralized monitoring in 43% of new studies starting in 2021 is encouraging but also highlights implementation of this component as a key area for RBM/RBQM growth.

### Reducing SDR/SDV

Centralized monitoring complements and supports reduced on-site SDR and SDV strategies. Although there is often hesitation in reducing SDR/SDV, we argue that this approach maximizes the benefits of more effective oversight mechanisms within RBM, such as remote and centralized monitoring. A landmark 2014 TransCelerate article concluded that SDV identifies only a small number of transcription errors and thus has a limited impact on trial data quality compared with a comprehensive RBM approach. Further support for RBM comes from a recent controlled study from Japan of trial sites implementing RBM that found partial SDV/SDR was associated with fewer data corrections compared with 100% SDV/SDR [13, 14].

On-site monitoring with the primary purpose of SDV is resource intensive. One study comparing RBM to extensive on-site monitoring found that the latter approach used more than twice the resources compared with the former [15]. In the Japanese study mentioned above, on-site monitoring time at sites implementing partial SDV/SDR was 30% less than time spent at sites performing 100% SDV/SDR. [13]

Despite evidence-based analysis demonstrating that it is less efficient and effective, and encouragement from regulators to employ strategic monitoring, 100% SDV remains ingrained in the clinical trial industry as the primary activity during on-site monitoring visits. This was reflected in a sub-analysis of our most recent survey data revealing that only 27% of trials started in 2021 implemented centralized monitoring with reduced SDV and/or reduced SDR (data not shown).

On-site, targeted SDV and SDR still has utility as part of a more comprehensive RBM-based strategy. RBQM supports reduced SDV/SDR through use of risk assessments to target data and processes critical to trial quality for on-site monitoring, thus increasing efficiency.

### Looking Down the Road—KRIs and QTLs

Typically defined by the trial sponsor and/or CRO and based on the risk assessment, KRIs and QTLs function as early warning signals activated by emerging threats to trial quality. These indicators signal operational teams to monitor specific data or processes more closely and, if necessary, take actions to mitigate any risks they pose to participant safety or data integrity. A major difference between these two components is that KRIs are typically measured at the site level to determine and inform site monitoring activities, while QTLs are higher-level indicators of overall trial quality.

KRI implementation may be underreported in our data set because these indicators are sometimes embedded in routine metrics (e.g., enrollment rate, screen fail rate, adverse event rate, etc.). Anecdotally, we have observed recent progress in using targeted KRIs to make informed decisions regarding monitoring activities. In contrast, QTLs are one component still on a learning curve with sponsors. Unlike the risk assessments that define them, uncertainty remains regarding regulator expectations around QTL implementation. Given the close relationship between the two components, we expect that once KRI adoption increases, sponsors may become more comfortable implementing QTLs.

### Study Limitations

This study does have some limitations. All trials included in the survey data set used CRO services, which may result in overrepresentation of certain therapeutic areas or trial types. Some trial sponsors did not outsource all RBQM activities; those activities done by the sponsor may not have been included in our data set. For example, a sponsor may conduct an initial risk assessment before contracting with a CRO, or the sponsor could decide to set and monitor QTLs on their own. Tracking capabilities differed between CROs participating in the survey; however, we have worked each year the survey has been conducted to achieve more consistency in tracking RBQM component implementation. In addition, our survey includes an undetermined number of “legacy” trials that were started before the release in 2016 of ICH E6(R2) guidance defining RBQM [12]. The design of these trials may not represent current best practices, and their inclusion may give the impression that RBM/RBQM adoption is increasing more slowly than it is. Although our results may underestimate the uptake of these practices, they are consistent year-over-year and provide valuable insight into adoption trends over time.

## Conclusion

An era of increasing trial decentralization, evolving study endpoints, new data collection modalities, and adaptive monitoring strategies requires new approaches to clinical trial design and management that recognize the importance of risk assessments and trial protocols focused on CtQ factors. RBQM is a flexible framework, not a one-size-fits-all approach. Although CROs expect that new trials include all or most RBQM components, not every trial needs to implement all eight. It is vital, however, that each clinical trial includes those components that optimize trial quality, considering the safety needs of the participants, the trial design, the technology used, and known threats to achieving the endpoints included in the trial.

In the 2021 update of ACRO’s RBM/RBQM landscape survey, we found that the vast majority of clinical trials in our data set included initial risk assessments, and almost as many trials included ongoing risk assessments. Although these findings are encouraging to those of us working to increase the adoption of RBM/RBQM, there remains much room for improvement. To realize the full potential of risk assessments, centralized monitoring, where possible, is needed to support site monitoring. Unfortunately, despite a 72% increase from 2020 to 2021, centralized monitoring was included in less than half of the ongoing trials in 2021. Increasing centralized monitoring is a logical next step toward improving trial quality through increased RBM adoption. Reducing SDV/SDR in conjunction with centralized monitoring will facilitate uptake and unlock the full potential of centralized monitoring. Continued support for risk assessments by regulatory bodies and trade organizations such as ACRO remains the core driver for the adoption and proper implementation of RBQM.

## Data Availability

The blinded and aggregated data that support the findings of this survey are available publicly at www.acrohealth.org and are available from the corresponding author, AA, upon reasonable request.
